# A Novel Indigoidine-like NRPS Gene from *Arthrobacter antioxidans* QL17 Enhances Oxidative Stress Resistance Through Radical Scavenging and Transcriptional Reprogramming

**DOI:** 10.3390/antiox15070846

**Published:** 2026-07-04

**Authors:** Xue Yu, Yujie Wu, Wei Zhang, Gaosen Zhang, Shiyu Wu, Xiaomin Niu, Liguo Yang, Qi Feng, Tuo Chen, Guangxiu Liu

**Affiliations:** 1 State Key Laboratory of Ecological Safety and Sustainable Development in Arid Lands, Northwest Institute of Eco-Environment and Resources, Chinese Academy of Sciences, Lanzhou 730000, China; yuxue@lzb.ac.cn (X.Y.); wuyujie@nieer.ac.cn (Y.W.); gaosenzhang@hotmail.com (G.Z.); niuxiaomin22@mails.ucas.ac.cn (X.N.); qifeng@lzb.ac.cn (Q.F.); liugx@lzb.ac.cn (G.L.); 2 Key Laboratory of Extreme Environmental Microbial Resources and Engineering of Gansu Province, Lanzhou 730000, China; wushiyu23@mails.ucas.ac.cn (S.W.); chentuo@lzb.ac.cn (T.C.); 3 State Key Laboratory of Cryospheric Science, Northwest Institute of Eco-Environment and Resources, Chinese Academy of Sciences, Lanzhou 730000, China; 4 Agronomy College, Gansu Agricultural University, Lanzhou 730000, China; yangliguo2001@126.com

**Keywords:** *Arthrobacter antioxidans* QL17, indigoidine-like NRPS, oxidative stress, radical scavenging, transcriptional reprogramming

## Abstract

Water-soluble blue microbial pigments with antioxidant activity remain rare, and their host-level protective mechanisms are poorly understood. Here, we identified the genetic basis of blue pigment biosynthesis in the glacier-derived strain *Arthrobacter antioxidans* QL17. Heavy-ion mutagenesis yielded a hyperpigmented mutant (M157) and a pigment-deficient mutant (M186), and pigment yield was positively associated with hydrogen peroxide (H_2_O_2_) tolerance. Genome mining identified MWM45_RS16760 as the sole core biosynthetic gene in a candidate nonribosomal peptide synthetase (NRPS)-like cluster. The encoded protein displayed an adenylation–peptidyl carrier protein–thioesterase (A-PCP-TE) architecture with a predicted L-glutamine-specific A domain, and its transcript abundance paralleled pigment production across the three strains. Phylogenetic analysis placed MWM45_RS16760 in a distinct actinomycete-associated indigoidine-like lineage separated from the characterized BpsA and IndC branches. Heterologous expression in *Escherichia coli* reconstructed a blue-pigment-producing phenotype, increased H_2_O_2_ tolerance, and was accompanied by enhanced extracellular DPPH and ABTS radical-scavenging activities in the culture supernatant. Comparative transcriptomics further revealed coordinated activation of oxidative-stress and proteostasis responses alongside repression of tryptophan biosynthesis and flagellar assembly. These findings identify MWM45_RS16760 as a candidate indigoidine-like NRPS associated with blue pigment biosynthesis and oxidative-stress resistance, with heterologous expression linked to enhanced radical scavenging and coordinated transcriptional reprogramming, expanding the phylogenetic and functional diversity of indigoidine-like systems.

## 1. Introduction

Aerobic metabolism inevitably generates reactive oxygen species (ROS), including superoxide (O_2_^−^), hydrogen peroxide (H_2_O_2_), and the hydroxyl radical (·OH). When their production outstrips cellular antioxidant defenses, oxidative stress carbonylates proteins, peroxidises lipids, and damages DNA, processes mechanistically linked to aging, neurodegeneration, cardiovascular disease, and cancer [1]. The same chemistry drives rancidity and shelf-life loss in foods, cosmetics, and pharmaceutical formulations, problems that industry has long managed with synthetic phenolic antioxidants such as butylated hydroxyanisole (BHA), butylated hydroxytoluene (BHT), and tert-butylhydroquinone (TBHQ). Accumulating evidence, however, links these additives to hepatic, renal, and neurological toxicity and to potential carcinogenicity, and several jurisdictions have begun to tighten their regulatory limits [2,3]. The search for safer, sustainable, and mechanistically well-defined natural alternatives has therefore become a research priority across food, biomedical, and cosmetic science.

Microorganisms have emerged as an attractive answer to this need. Compared with plant-derived antioxidants, microbial pigments can be produced through scalable fermentation, are independent of seasonal supply, and can be tuned by genetic or process engineering to meet defined specifications [4]. Representative bacterial pigments such as violacein from *Chromobacterium violaceum*, prodigiosin from *Serratia marcescens*, and carotenoids from actinomycetes consistently display potent DPPH and ABTS scavenging activity and inhibit lipid peroxidation [5,6]. Yet the antioxidant pigments characterized to date are overwhelmingly red, purple, orange, or yellow, leaving stable, water-soluble blue bacterial pigments comparatively rare [7]. This gap is more than aesthetic: the extended π-conjugation systems responsible for blue absorption typically correspond to lower HOMO-LUMO energy gaps and higher electron-donating capacity than those of shorter-wavelength chromophores, properties that are electrochemically favorable for radical scavenging and electron-transfer reactions [8]. Furthermore, the bipyridyl skeleton of indigoidine supports both sequential electron transfer (SET) and hydrogen atom transfer (HAT) mechanisms in radical scavenging [9], distinguishing it from shorter-conjugation pigments whose antioxidant activity may rely primarily on single-electron donation. Most mechanistic studies of pigment-mediated protection rest on cell-free radical-scavenging assays, and few examine whether bacterial pigments also reshape host transcriptional networks responsible for oxidative defense—leaving an important dimension of ‘natural antioxidant action’ unexplored.

Among candidate blue pigments, indigoidine (5,5′-diamino-4,4′-dihydroxy-3,3′-diazadiphenoquinone-(2,2′)) is particularly compelling. Its bipyridyl skeleton, formed by oxidative dimerisation of two L-glutamine molecules, supports an extended π-conjugation and an intermolecular hydrogen-bonding network that confer striking light, pH, and thermal stability together with the redox activity expected of an antioxidant [7,8]. Biosynthetically, indigoidine is assembled by a single-module nonribosomal peptide synthetase (NRPS) with a canonical adenylation-oxidation-peptidyl carrier protein-thioesterase (A-Ox-PCP-TE) architecture. The archetypal enzyme BpsA from *Streptomyces lavendulae* and its enterobacterial homolog IndC from *Dickeya* spp. have been characterized structurally and biochemically and successfully reconstituted in *Escherichia coli* and *Saccharomyces cerevisiae* [10,11,12]. Indigoidine has been linked to antioxidant, antimicrobial, cryoprotective, and UV-shielding roles [13,14]; nevertheless, quantitative and mechanistic evaluation of its antioxidant function-particularly at the level of host transcriptional regulation-has remained limited.

Actinomycetes are renowned reservoirs of NRPS- and PKS-derived natural products, but a substantial fraction of their biosynthetic gene clusters (BGCs) remain cryptic under standard cultivation [15]. Genome-mining platforms such as antiSMASH, coupled with heterologous expression in minimal-background chassis, have become essential for accessing this hidden chemistry [16]. To date, characterized indigoidine synthases are almost entirely confined to *Streptomyces* and *Dickeya/Erwinia* lineages; their distribution and function in non-streptomycete actinomycetes such as *Arthrobacter* are largely undefined, despite the widespread occurrence of pigmented *Arthrobacter* isolates in glaciers, alpine soils, and other extreme habitats [17]. Of particular relevance, *A. antioxidans* QL17, isolated from the East Rongbuk Glacier of Mount Everest, secretes a water-soluble blue pigment and tolerates up to 400 mM H_2_O_2_ [18]; the same strain also produces the antioxidant carotenoid arthroxanthin [19], a terpenoid pigment biosynthesised through a mevalonate/MEP-independent isoprenoid pathway that is genetically and biochemically distinct from the NRPS-based blue pigment system investigated here. The co-occurrence of two structurally unrelated antioxidant pigment systems marks *A. antioxidans* QL17 as a particularly promising chassis for the discovery of new antioxidant biosynthetic machinery. The genetic basis of its blue pigmentation, however, has not been resolved.

In this study, we report the identification and functional characterization of MWM45_RS16760, a novel indigoidine-like nonribosomal peptide synthetase (NRPS) gene from *A. antioxidans* QL17. Using pigment-overproducing and pigment-deficient mutants generated by heavy-ion beam mutagenesis [20], we establish a positive correlation between blue-pigment yield and H_2_O_2_ tolerance and identify RS16760 as the underlying biosynthetic gene through genome mining and RT-qPCR. Phylogenetic analysis places the encoded A-PCP-TE enzyme within a distinct actinomycete-associated clade divergent from the reference indigoidine synthases BpsA and IndC. Heterologous expression in *E. coli* reconstitutes blue-pigment biosynthesis and significantly enhances H_2_O_2_ tolerance and free-radical scavenging activity. Comparative transcriptomics further reveals concurrent activation of canonical oxidative stress defense pathways and coordinated reprogramming of proteostasis, amino-acid metabolism, and motility pathways. Collectively, these findings establish MWM45_RS16760 as a dual-function indigoidine-like NRPS that enhances oxidative stress resistance through direct radical scavenging and systemic transcriptional reprogramming, providing a genetic target for engineering antioxidant-enhanced microorganisms.

## 2. Materials and Methods

### 2.1. Bacterial Strains, Plasmids, and Culture Conditions

*Arthrobacter antioxidans* QL17, originally isolated from the East Rongbuk Glacier of Mt. Everest, is deposited at the Guangdong Microbial Culture Collection Center (GDMCC 1.2948^T^) and the Japan Collection of Microorganisms (JCM 35246^T^) [18]. Two derivatives used in this study, the pigment-overproducing mutant M157 and the pigment-deficient mutant M186, were generated from QL17 by carbon-ion irradiation as described below *Escherichia coli* BL21(DE3) (C2527H, New England Biolabs, Ipswich, MA, USA) was used as the heterologous expression host, and pET41a (+) (V010975, NovoPro Bioscience, Shanghai, China) was used as the expression vector.

All *A. antioxidans* strains were maintained on R_2_A agar and cultured in R_2_A broth at 15 °C with shaking at 160 rpm. *E. coli* strains were maintained on Luria–Bertani (LB) agar and cultured in LB broth at 37 °C with shaking at 180 rpm. Kanamycin was added at 50 μg/mL for selection of pET41a-derived transformants. Glycerol stocks were stored at −80 °C in 20% (*v*/*v*) glycerol.

### 2.2. Heavy-Ion Irradiation and Screening of Pigment Mutants

Exponentially growing cells of *A. antioxidans* QL17 were harvested, washed twice with sterile 0.9% (*w*/*v*) NaCl, and resuspended in 0.9% NaCl to OD_600_ = 1.0. One milliliter of the cell suspension was irradiated with carbon ions (^12^C^6+^, 80 MeV/u) at a single dose of 500 Gy (dose rate, 20 Gy/min) at the Heavy Ion Research Facility in Lanzhou (HIRFL), Institute of Modern Physics, Chinese Academy of Sciences. The irradiation dose of 500 Gy was selected based on preliminary survival assays indicating approximately 20–30% colony-forming survival for *A. antioxidans* QL17 at this dose, a range considered optimal for maximizing mutagenic diversity while ensuring adequate population recovery for screening [21,22]. An unirradiated aliquot from the same batch served as the control.

After irradiation, cells were diluted and spread on R_2_A agar plates, followed by incubation at 15 °C for 7 days. A total of 1056 colonies were picked into 96-deep-well plates containing R_2_A broth and cultured at 15 °C and 160 rpm for 3 days. The absorbance of each fermentation broth at 590 nm was measured using a SpectraMax 190 microplate reader (Molecular Devices, San Jose, CA, USA), and mutants displaying the highest and lowest A_590_ values were selected as M157 and M186, respectively. Genetic stability of the selected mutants was verified by five successive subcultures.

### 2.3. Pigment Quantification by UV-Vis Spectroscopy

The wild-type, M157, and M186 strains were cultured in R_2_A broth at 15 °C and 160 rpm for 3 days. Cultures were centrifuged at 5000 rpm for 5 min, and the clarified supernatants were used for spectroscopic analysis. Aliquots (200 μL) were transferred into clear 96-well plates, and absorption spectra were recorded from 350 to 750 nm using a SpectraMax 190 microplate reader. Sterile R_2_A medium processed in parallel was used as the blank. Three independent biological replicates were analyzed.

### 2.4. H_2_O_2_ Tolerance Assays

#### 2.4.1. H_2_O_2_ Tolerance Assay for *A. antioxidans* QL17

The wild-type, M157, and M186 strains were grown in R_2_A broth at 15 °C and 160 rpm to OD_600_ = 1.0. Cells were harvested, washed twice with sterile 0.9% NaCl, and resuspended in 0.9% NaCl to the original OD_600_. Aliquots of the suspensions were challenged with freshly prepared H_2_O_2_ at final concentrations of 50, 100, 200, 300, 400, 500, and 600 mM at 15 °C for 120 min. Untreated suspensions received an equal volume of sterile 0.9% NaCl. After treatment, samples were immediately serially diluted in 0.9% NaCl, plated on R_2_A agar, and incubated at 15 °C for 7 days. Colony-forming units (CFUs) were counted, and survival rates were calculated as: Survival rate (%) = CFU_treated_/CFU_untreated_ × 100%. Three independent biological replicates were performed.

#### 2.4.2. H_2_O_2_ Tolerance Assay for Recombinant *E. coli*

*E. coli* BL21(DE3) harboring pET41a (empty vector) or pET41a-RS16760 was grown overnight in LB-kanamycin at 37 °C and 180 rpm, subcultured (1:100, *v*/*v*) into fresh medium, and grown to OD_600_ = 0.6. Expression was induced with 200 μM IPTG at 18 °C for 20 h. Induced cells were harvested, washed twice with sterile 0.9% NaCl, and resuspended to OD_600_ = 1.0. Cell suspensions were treated with H_2_O_2_ at final concentrations of 0, 5, 10, 20, 30, 40, and 50 mM at 18 °C for 120 min. Surviving cells were quantified by CFU counting on LB-kanamycin agar after incubation at 37 °C for 16 h. Survival rates were calculated as described above. Three independent biological replicates were performed.

Overall H_2_O_2_ tolerance was quantified by calculating the area under the survival curve (AUC) in GraphPad Prism. Survival rate (%) was plotted against H_2_O_2_ concentration (0–600 mM), and AUC was calculated using the trapezoidal method with Y = 0 as the baseline. AUC values from three biological replicates were compared by one-way ANOVA followed by Tukey’s test.

### 2.5. Genome Mining and Analysis of the NRPS-like Gene Cluster

Secondary metabolite biosynthetic gene clusters in the *A. antioxidans* QL17 genome (GenBank accession CP095501) were predicted using antiSMASH 7.0 with relaxed detection strictness and otherwise default parameters. Among the predicted clusters, a NRPS-like cluster containing a single core biosynthetic gene, MWM45_RS16760 (RefSeq protein accession WP_247827427.1), was selected for further analysis. Domain organization and adenylation-domain substrate preference were predicted using the integrated NRPSPredictor2 module [23]. Cluster organization was visualized based on the antiSMASH output and refined in Adobe Illustrator.

### 2.6. Protein Structure Modeling, Structural Comparison, and Molecular Docking

#### 2.6.1. Structure Prediction and Structural Superposition

The full-length protein sequence encoded by MWM45_RS16760 (1150 aa) was submitted to the AlphaFold Server for structure prediction using default settings [24]. The best-ranked model was selected for downstream analysis. The amino acid sequence of the reference indigoidine synthase BpsA from *Streptomyces lavendulae* [25] (UniProt accession Q1MWN4) was modeled using the same pipeline. Structural superposition between RS16760 and BpsA was performed in PyMOL, and structural similarity was evaluated using TM-align [26].

#### 2.6.2. Molecular Docking with L-Glutamine

To investigate the predicted substrate specificity of the A domain for L-glutamine (L-Gln), molecular docking was performed using AutoDock Vina v1.2 [27]. The full-length AlphaFold2 model of MWM45_RS16760 was used as the receptor; non-protein atoms were removed using BioPython (v1.83), and polar hydrogens and Gasteiger partial charges were assigned using Open Babel (v3.1) [24]. The three-dimensional structure of L-Gln was retrieved from PubChem (CID 5961) and processed identically. A conformer was generated using the ETKDGv3 algorithm in RDKit with MMFF94 optimization.

The docking search space was defined as a cubic grid box of 25 × 25 × 25 Å, centered at coordinates (−31.89, −11.42, −2.05) Å, corresponding to the substrate-binding pocket identified by PARAS analysis (residues 195–315). Docking was performed with an exhaustiveness of 16, generating 10 independent binding poses per run. To assess reproducibility, docking was independently repeated three times using different random seeds (42, 123, and 2024). To evaluate substrate selectivity, five additional amino acids (L-glutamate, L-asparagine, L-lysine, L-alanine, and L-phenylalanine) were docked under identical conditions. Hydrogen bonds between L-Gln and active-site residues were visualized in PyMOL (v2.6).

### 2.7. Phylogenetic Analysis of NRPS Adenylation Domains

Homologous NRPS proteins were retrieved by BLASTp against the NCBI non-redundant protein database using the RS16760 protein as the query. Redundant sequences were removed using CD-HIT [28]. Conserved adenylation domains were extracted using HMMER [29] against the Pfam AMP-binding profile (PF00501) [30]. Two experimentally characterized indigoidine synthases, BpsA from Streptomyces lavendulae and IndC from Dickeya fangzhongdai [31], were included as references. Adenylation-domain sequences were aligned using MAFFT [32] and trimmed using trimAl [33]. A maximum-likelihood phylogenetic tree was inferred using IQ-TREE [34] with branch support estimated by SH-aLRT and ultrafast bootstrap analyses (1000 replicates each). The tree was visualized using iTOL [35].

### 2.8. RNA Extraction and RT-qPCR Analysis of RS16760 Expression

The wild-type, M157, and M186 strains were cultured in R_2_A broth at 15 °C and 160 rpm and harvested at OD_600_ = 1.0. Total RNA was extracted using the CTAB method, followed by DNase I treatment to remove genomic DNA. First-strand cDNA was synthesized using random hexamer primers. Quantitative PCR was performed on an ABI 7300 Real-Time PCR System (Applied Biosystems, Carlsbad, CA, USA) using ChamQ SYBR Color qPCR Master Mix (Q411, Vazyme Biotech, Nanjing, China). The cycling program consisted of 95 °C for 30 s, followed by 40 cycles of 95 °C for 10 s and 60 °C for 30 s, with melt-curve analysis at the end. The 16S rRNA gene was used as the reference gene for RT-qPCR normalization, as all three strains were cultured under identical, non-stress conditions; this gene has been validated as a stable reference for comparative expression analysis in actinobacteria under standard growth conditions. Relative expression levels were calculated using the 2^−ΔΔCt^ method [36], with the wild-type as the calibrator. Primer sequences are listed in Supplementary Table S1. Three biological replicates with three technical replicates each were analyzed.

### 2.9. Construction of pET41a-RS16760 and Heterologous Expression in E. coli

The full-length coding sequence of MWM45_RS16760 was chemically synthesized without codon optimization by Shanghai Xinzhuo Biotechnology Co., Ltd. (Shanghai, China) and cloned into pET41a (+) to generate pET41a-RS16760. The construct was verified by colony PCR and Sanger sequencing. The recombinant plasmid and the empty vector were transformed into chemically competent *E. coli* BL21(DE3) cells by heat shock and selected on LB-kanamycin agar. For pigment production, single colonies were grown overnight in LB-kanamycin, subcultured (1:100, *v*/*v*) into fresh medium, and grown at 37 °C to OD_600_ = 0.6. Expression was induced with 200 μM IPTG at 18 °C for 20 h. Pigment accumulation was assessed visually in the cell pellet and culture supernatant after centrifugation.

### 2.10. DPPH and ABTS Radical-Scavenging Assays

Culture supernatants from *E. coli* pET41a and *E. coli* pET41a-RS16760 were prepared after 20 h of IPTG induction as described above. Supernatants were clarified by centrifugation, passed through 0.22-μm filters, and used directly for radical-scavenging assays. Sterile LB medium processed in parallel was used as the blank.

DPPH radical-scavenging activity was measured using a commercial kit (BC4750, Solarbio Life Sciences, Beijing, China), and absorbance was recorded at 515 nm. ABTS radical-scavenging activity was measured using a commercial kit (BC4770, Solarbio Life Sciences, Beijing, China), and absorbance was recorded at 405 nm. For both assays, the intrinsic absorbance of the blue supernatant was subtracted using a supernatant-only control. Radical-scavenging rates were calculated according to the manufacturer’s instructions. Three biological replicates with three technical replicates each were analyzed.

### 2.11. Transcriptome Sequencing and Differential Expression Analysis

After 20 h of IPTG induction, *E. coli* pET41a and *E. coli* pET41a-RS16760 cells were harvested by centrifugation, immediately frozen in liquid nitrogen, and stored at −80 °C until RNA extraction. Based on the bacterial strand-specific RNA-seq workflow provided by Majorbio, total RNA was extracted using the CTAB method, genomic DNA was removed, and high-quality RNA was used for strand-specific library preparation. Paired-end sequencing was performed on an Illumina NovaSeq 6000 platform. The same workflow indicates that clean reads were mapped with Bowtie2 [37], and transcript abundance was quantified with RSEM [38].

In the present study, clean reads were aligned to the reference genome of the heterologous host, *E. coli* BL21(DE3) (GCA_013167015.1), and differential expression analysis between *E. coli* pET41a-RS16760 and the empty-vector control was performed using DESeq2 [39]. *p*-values were corrected for multiple testing using the Benjamini–Hochberg false discovery rate (FDR) method, and genes with FDR-adjusted *p*-value ≤ 0.05 and |log2 fold change| ≥ 1 were considered differentially expressed. GO and KEGG enrichment analyses were performed using clusterProfiler v4.14.6 in R v4.4.0 [40], with adjusted *p*-value < 0.05 considered significant. Volcano plots, enrichment bubble plots, and heatmaps were generated using ggplot2 v4.0.1, ggrepel v0.9.6, and pheatmap v1.0.13 in R v4.4.0, respectively.

### 2.12. Statistical Analysis

Unless otherwise stated, experiments were performed with three independent biological replicates. Data are presented as mean ± standard deviation (SD). Statistical analyses were performed using GraphPad Prism 10. Comparisons between two groups were analyzed using a two-tailed unpaired Student’s *t*-test. Comparisons among three or more groups were analyzed using one-way ANOVA followed by Tukey’s post hoc test. Experiments involving two independent variables were analyzed using two-way ANOVA followed by Tukey’s post hoc test. Differences with *p* < 0.05 were considered statistically significant.

## 3. Results

### 3.1. Blue Pigment Production Is Positively Associated with Oxidative Stress Tolerance in Arthrobacter Antioxidans QL17

**Figure 1:**
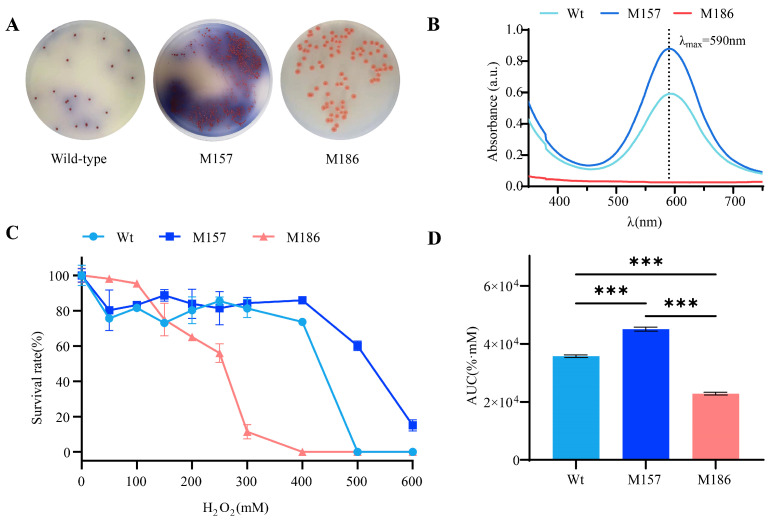
Blue pigment production is positively associated with oxidative stress tolerance in *A. antioxidans* QL17 and its heavy-ion-induced mutants. (**A**) Pigmentation phenotypes of the wild-type strain (Wild-type), the pigment-overproducing mutant M157, and the pigment-deficient mutant M186 on R_2_A agar plates. (**B**) UV-Vis absorption spectra (350–750 nm) of the fermentation broths of the wild-type (Wt, cyan), M157 (blue), and M186 (red). (**C**) Survival rates of the wild-type, M157, and M186 under increasing concentrations of H_2_O_2_ (0–600 mM). Data are presented as mean ± SD of three independent biological replicates. (**D**) Area under the survival curve (AUC, %·mM) calculated from panel C for each strain. Statistical significance was assessed by one-way ANOVA followed by Tukey’s post hoc test. *** *p* < 0.001. Data are presented as mean ± SD of three independent biological replicates.

To investigate whether blue pigment production in *A. antioxidans* QL17 is associated with oxidative stress resistance, two pigmentation-altered mutants were obtained by heavy-ion irradiation (carbon ion, ^12^C^6+^, 80 MeV/u) and screened from 1056 mutant colonies based on absorbance at 590 nm. Mutant M157 exhibited markedly enhanced blue pigmentation, whereas mutant M186 displayed a complete loss of the blue phenotype on R_2_A agar (Figure 1A), providing a panel of strains with contrasting pigment phenotypes for subsequent functional comparison.

UV-Vis absorption spectra (350–750 nm) of the fermentation broths further confirmed these differences. The wild-type and M157 both exhibited a well-defined absorption maximum at 590 nm, consistent with the characteristic absorption peak of indigoidine-type blue pigments [25]. The absorbance at 590 nm in M157 was approximately 1.5 times that of the wild-type. In contrast, M186 remained near baseline across the visible range, with no distinct absorption peak detectable at 590 nm (Figure 1B). The screening wavelength of 590 nm corresponds to the characteristic absorption maximum of indigoidine-type blue pigments in aqueous solution, a diagnostic feature consistently reported across diverse bacterial producers [11,25,31,41]. The identity of the spectral profile (λ_max_ = 590 nm) across the wild-type and M157, confirmed by full UV-Vis scanning (Figure 1B), indicates that A_590_-based screening reflected differences in pigment concentration rather than changes in chromophore identity.

H_2_O_2_ tolerance of the three strains was then evaluated across 50–600 mM. At 50–250 mM, all three strains retained survival rates above approximately 55%, although strain-dependent differences became increasingly apparent with rising oxidant concentrations. M186 showed a pronounced loss of viability at 300 mM and no detectable survival at ≥400 mM, whereas the wild-type retained appreciable viability at 400 mM but showed no detectable survival at 500–600 mM. In contrast, M157 maintained 60% survival at 500 mM and 15–17% survival at 600 mM, the highest concentration tested (Figure 1C). To quantify the overall oxidative stress tolerance of each strain, the area under the survival curve (AUC, %·mM) was calculated by integrating survival rate over the full H_2_O_2_ concentration range (0–600 mM). One-way ANOVA followed by Tukey’s post hoc test revealed that AUC values differed significantly among all three strains (*p* < 0.001 for all pairwise comparisons), with the rank order M157 > wild-type > M186 (Figure 1D). This rank order was perfectly concordant with the rank order of pigment yield (Figure 1B), providing quantitative statistical support for a positive association between blue pigment production and oxidative stress tolerance in *A. antioxidans* QL17. These findings prompted us to investigate the genetic basis underlying pigment biosynthesis.

### 3.2. Genome Mining and Structural Analyses Support MWM45_RS16760 as a Candidate Indigoidine-like NRPS in A. antioxidans QL17

**Figure 2:**
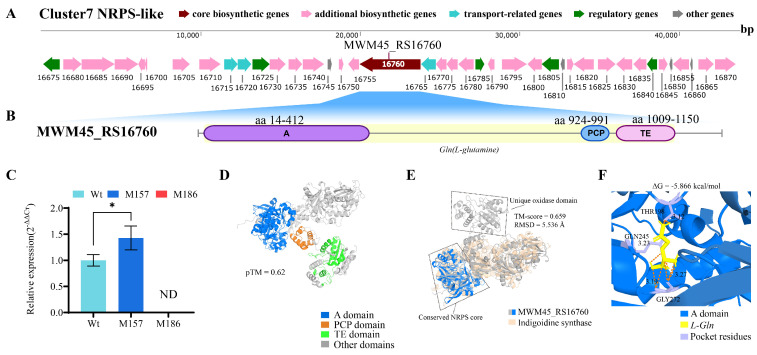
Genome mining and structural characterization identify MWM45_RS16760 as a core NRPS-like indigoidine synthetase whose expression correlates with the blue pigment phenotype. (**A**) Genomic organization of the Cluster 7 NRPS-like biosynthetic gene cluster identified in *A. antioxidans* QL17. Genes are color-coded by predicted function: core biosynthetic genes (dark red), additional biosynthetic genes (pink), transport-related genes (cyan), regulatory genes (green), and other genes (grey). (**B**) Domain architecture of the MWM45_RS16760-encoded protein, displaying a canonical A-PCP-TE modular organization. The adenylation (A) domain is predicted to specifically activate L-glutamine (L-Gln). (**C**) Relative transcript abundance of MWM45_RS16760 in the wild-type (Wt), M157, and M186 strains determined by RT-qPCR. Data are presented as mean ± SD of three independent biological replicates, each performed in technical triplicate. Statistical significance was assessed using Student’s *t*-test (* *p* < 0.05). ND, not detected. (**D**) Predicted three-dimensional structure of the MWM45_RS16760 protein modeled using AlphaFold2 (pTM = 0.62), with the A domain (blue), PCP domain (orange), and TE domain (green) highlighted. (**E**) Structural superposition of the MWM45_RS16760 model (blue/grey) with the reference indigoidine synthase BpsA from Streptomyces lavendulae (wheat). TM-score = 0.659, RMSD = 5.536 Å. (**F**) Close-up view of the molecular docking between the A domain of MWM45_RS16760 (blue cartoon) and L-glutamine (L-Gln, yellow sticks) with a predicted binding free energy of ΔG = −5.834 kcal/mol. Key residues forming the substrate-binding pocket (purple sticks) and hydrogen bonds (orange dashed lines, distances in Å) are indicated. The docking pose shown represents the lowest-energy conformation from triplicate independent runs (seeds 42, 123, and 2024; pairwise pose RMSD = 0.351–0.599 Å; see Supplementary Table S2 for the complete multi-ligand comparison).

To explore the genetic basis of blue pigment biosynthesis, the whole-genome sequence of *A. antioxidans* QL17 was analyzed using antiSMASH 7.0. A non-ribosomal peptide synthetase (NRPS)-like biosynthetic gene cluster, designated Cluster 7, was identified, in which MWM45_RS16760 was the sole gene annotated as a core biosynthetic enzyme (Figure 2A). Domain architecture prediction by the integrated NRPSPredictor2 module revealed a canonical A-PCP-TE modular organization, with the adenylation (A) domain spanning aa 14-412 predicted to recognize L-glutamine (L-Gln) as the most likely substrate (Figure 2B), consistent with the substrate preferences reported for indigoidine synthases.

RT-qPCR analysis was then performed to examine whether the transcript level of MWM45_RS16760 was associated with the pigment phenotype across the three strains. Relative to the wild-type, MWM45_RS16760 transcript abundance was significantly elevated in M157 (*p* < 0.05), whereas no transcript was detectable in M186 (Figure 2C), consistent with a gene-inactivating mutation at or upstream of the RS16760 locus; the precise nature of this genomic alteration (e.g., promoter disruption, gene deletion, or structural rearrangement) awaits characterization by whole-genome re-sequencing of the mutant strain. This expression pattern was consistent with the pigment yield pattern observed in Figure 1, providing independent transcriptional evidence linking MWM45_RS16760 to blue pigment production.

To further evaluate the structural plausibility of MWM45_RS16760 as an indigoidine-like NRPS, its three-dimensional structure was predicted using AlphaFold2 (pTM = 0.62), yielding a plausible multidomain architecture comprising the A, PCP, and TE domains (Figure 2D). Structural superposition with the reference indigoidine synthase BpsA from *Streptomyces lavendulae* revealed a conserved NRPS catalytic core (TM-score = 0.659, RMSD = 5.536 Å), while MWM45_RS16760 contained an additional inserted region absent in BpsA, suggesting architectural divergence (Figure 2E). Molecular docking of L-Gln into the A-domain pocket, validated by triplicate independent runs (ΔG = −5.858 ± 0.014 kcal/mol, *n* = 3; Supplementary Table S2), yielded an optimal binding free energy of ΔG = −5.866 kcal/mol, with four polar contacts formed between the substrate and Thr198, Gln245, and Gly272 (Figure 2F). Comparative docking of five additional amino acids confirmed that L-Gln achieved the highest number of pocket-specific contacts and a significantly more favorable binding affinity than its closest structural analog, L-glutamate (ΔG = −5.720 ± 0.020 kcal/mol; Welch’s *t*-test, *p* = 0.001; Supplementary Table S2), consistent with the predicted L-Gln preference of the A domain. Taken together, these results support MWM45_RS16760 as a strong candidate indigoidine-like NRPS associated with blue pigment biosynthesis in *A. antioxidans* QL17 and justify its direct functional validation.

### 3.3. Adenylation-Domain Phylogeny Places MWM45_RS16760 in a Distinct Actinomycete-Associated Indigoidine-like Lineage

**Figure 3:**
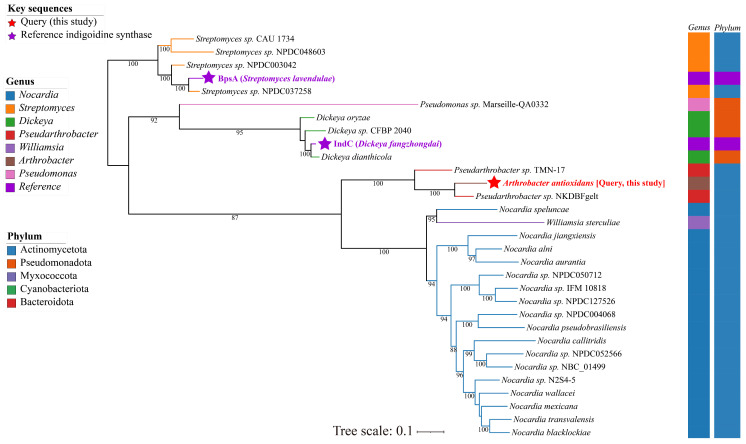
Maximum-likelihood phylogenetic analysis of the adenylation (A) domain places MWM45_RS16760 within a distinct actinomycete-associated indigoidine-like NRPS clade. A maximum-likelihood phylogenetic tree was reconstructed from the A domain sequences of MWM45_RS16760 (*A. antioxidans* QL17) and bacterial homologs retrieved from the NCBI nr database, together with two reference indigoidine synthases: BpsA from *Streptomyces lavendulae* and IndC from *Dickeya fangzhongdai*. The query sequence is marked with a red star, and the two reference indigoidine synthases are marked with purple stars. Ultrafast bootstrap (UFBoot) support values (≥80%) are shown at internal nodes. The inner and outer colored strips annotate taxonomic affiliation at the genus and phylum levels, respectively. The tree scale bar represents 0.1 amino acid substitutions per site. For brevity, only a representative subset of the 371 taxa is shown; the complete tree is provided in Supplementary Figure S1.

To place MWM45_RS16760 in an evolutionary context, a maximum-likelihood phylogenetic tree was reconstructed using the adenylation (A) domain sequence of MWM45_RS16760, 370 bacterial homologs retrieved from the NCBI non-redundant protein database, and the two well-characterized reference indigoidine synthases BpsA from *Streptomyces lavendulae* and IndC from *Dickeya fangzhongdai* (Figure 3). The complete tree is shown in Supplementary Figure S1.

The phylogeny recovered several well-supported subclades broadly consistent with taxonomic affiliation. BpsA grouped within a *Streptomyces*-dominated subclade (Actinomycetota), whereas IndC fell within a *Dickeya*-dominated subclade (Pseudomonadota), in agreement with their known taxonomic origins. The A domain of MWM45_RS16760 clustered with homologs from *Pseudarthrobacter* spp. (strains TMN-17 and NKDBFgelt) within a strongly supported actinomycete-associated subclade (UFBoot = 100), which was clearly separated from both the BpsA-containing *Streptomyces* clade and the IndC-containing Dickeya clade.

This subclade was further nested within a broader group dominated by *Nocardia* spp. and other members of the phylum Actinomycetota, suggesting that MWM45_RS16760 and its closest homologs form a distinct actinomycete-associated lineage related to, but separate from, the BpsA and IndC lineages. To our knowledge, no experimentally characterized indigoidine synthase has yet been reported from this *Pseudarthrobacter*/*Arthrobacter*-associated subclade, suggesting that this lineage remains functionally underexplored.

Taken together, these results place the A domain of MWM45_RS16760 in a distinct actinomycete-associated indigoidine-like lineage, providing an evolutionary framework for its subsequent functional characterization.

### 3.4. Heterologous Expression of MWM45_RS16760 in Escherichia coli Reconstitutes a Blue-Pigment-Producing Phenotype and Enhances Oxidative Stress Tolerance and Radical-Scavenging Activity

**Figure 4:**
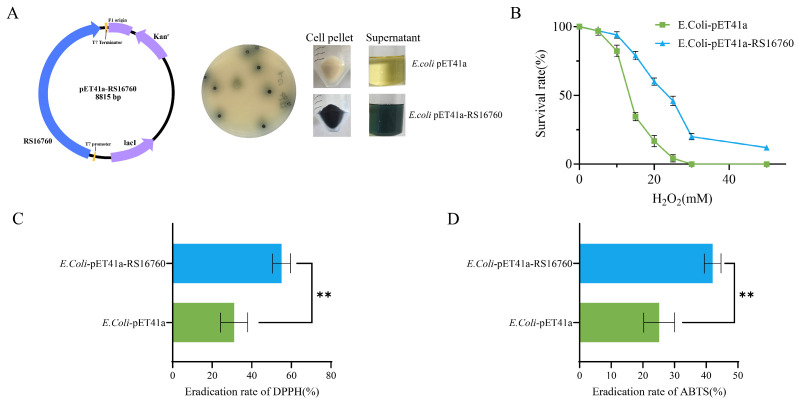
Heterologous expression of MWM45_RS16760 in *E. coli* reconstitutes blue pigment biosynthesis and confers enhanced H_2_O_2_ tolerance and free-radical scavenging activity. (**A**) Schematic map of the recombinant expression plasmid pET41a-RS16760 (8815 bp; left), the corresponding kanamycin-resistant transformants on LB agar (middle), and the pigmentation phenotypes of cell pellets and culture supernatants of *E. coli* pET41a (top) and *E. coli* pET41a-RS16760 (bottom) after 20 h of IPTG induction (right). (**B**) H_2_O_2_ tolerance of the empty-vector control *E. coli* pET41a (green) and the recombinant H_2_O_2_ pET41a-RS16760 (blue). Data are presented as mean ± SD of three independent biological replicates. (**C**,**D**) DPPH (**C**) and ABTS (**D**) free-radical scavenging activities of culture supernatants from *E. coli* pET41a (green) and *E. coli* pET41a-RS16760 (blue). Data are presented as mean ± SD of three independent biological replicates. Statistical significance was assessed by Student’s *t*-test (** *p* < 0.01).

To directly test the functional role of MWM45_RS16760, the gene was cloned into pET41a and heterologously expressed in *E. coli* BL21(DE3) (Figure 4A, left). Kanamycin-resistant transformants were obtained on LB agar (Figure 4A, middle). After IPTG induction, *E. coli* pET41a-RS16760 accumulated a deep blue color in both the cell pellet and the culture supernatant, whereas the empty-vector control *E. coli* pET41a remained colorless (Figure 4A, right), demonstrating that MWM45_RS16760 alone is sufficient to reconstruct a blue-pigment-producing phenotype in a heterologous host.

The H_2_O_2_ tolerance of the two recombinant strains was then evaluated across 0–50 mM. Both strains retained near-complete viability at 0–5 mM, but their survival curves diverged sharply at higher concentrations. The empty-vector control *E. coli* pET41a lost most of its viability between 10 and 25 mM and was virtually nonviable at ≥30 mM. In contrast, *E. coli* pET41a-RS16760 retained substantially higher viability throughout the entire concentration range. At 20 mM H_2_O_2_, *E. coli* pET41a-RS16760 retained approximately 60% survival compared with approximately 16.8% for the empty-vector control (∼3.6-fold difference). At 25 mM, the RS16760-expressing strain maintained approximately 45% survival whereas the control showed less than 5% viability. At the highest concentration tested (50 mM), *E. coli* pET41a-RS16760 retained 12% survival while the control was essentially non-viable (Figure 4B), indicating that heterologous expression of MWM45_RS16760 markedly enhances H_2_O_2_ tolerance in *E. coli*.

The radical-scavenging activity of culture supernatants was further assessed using DPPH and ABTS assays. The culture supernatant of *E. coli* pET41a-RS16760 showed DPPH and ABTS radical-scavenging rates of approximately 55% and 42%, respectively, both significantly higher than those of the empty-vector control (* *p* < 0.01; Figure 4C,D), indicating that the pigment-producing recombinant strain possesses enhanced extracellular radical-scavenging activity. DPPH and ABTS radical-scavenging activities are reported as percentage scavenging rates of unfractionated culture supernatants and therefore represent bulk extracellular antioxidant capacity rather than the specific activity of a purified compound. Normalization to pigment concentration and IC_50_ determination will require purification of the responsible chromophore.

Taken together, these results provide direct functional evidence that heterologous expression of MWM45_RS16760 is sufficient to reconstruct a blue-pigment-producing phenotype and to enhance oxidative stress tolerance and extracellular radical-scavenging activity in *E. coli*.

### 3.5. Comparative Transcriptomics Reveals Coordinated Transcriptional Reprogramming Associated with MWM45_RS16760 Expression

**Figure 5:**
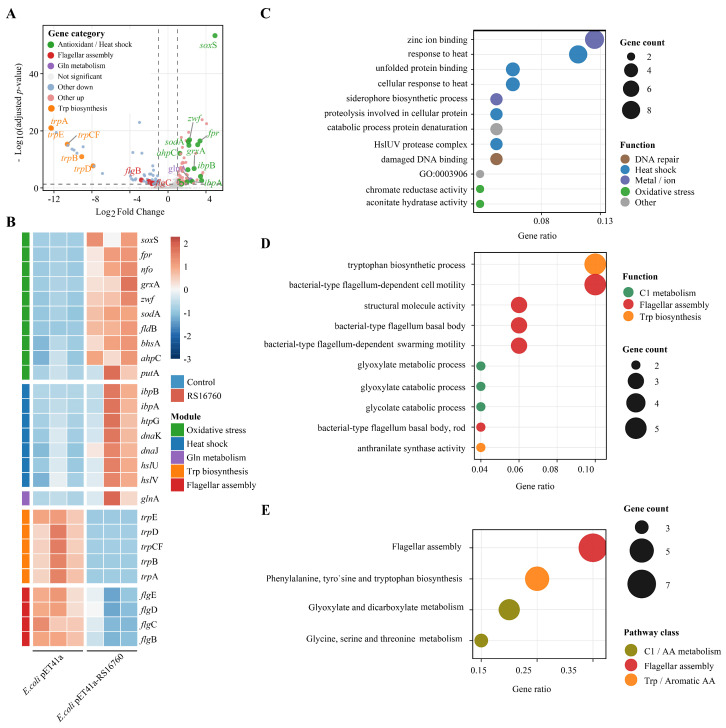
Comparative transcriptomics reveals a four-pronged transcriptional reprogramming in *E. coli* upon heterologous expression of MWM45_RS16760. (**A**) Volcano plot of differentially expressed genes (DEGs) between *E. coli* pET41a-RS16760 and the empty-vector control *E. coli* pET41a. Dashed lines indicate the significance thresholds (|log_2_fold change| ≥ 1 and adjusted *p*-value ≤ 0.05). Genes are colored by functional category: antioxidant/heat-shock response (green), flagellar assembly (red), Gln metabolism (purple), Trp biosynthesis (orange), other up-regulated (pink), other down-regulated (light blue), and non-significant (grey). (**B**) Heatmap of 27 representative DEGs grouped into five functional modules: oxidative stress response (green), heat shock/proteostasis (blue), Gln metabolism (purple), Trp biosynthesis (orange), and flagellar assembly (red). Columns represent three independent biological replicates for *E. coli* pET41a (Control, left) and *E. coli* pET41a-RS16760 (RS16760, right). (**C**,**D**) Gene Ontology (GO) enrichment analysis of up-regulated (**C**) and down-regulated (**D**) DEGs, covering biological process, molecular function, and cellular component ontologies. Bubble size is proportional to gene count and color indicates functional category as labeled. The x-axis shows the gene ratio. (**E**) KEGG pathway enrichment analysis of down-regulated DEGs. Up-regulated DEGs did not yield statistically significant KEGG pathway enrichment under these criteria and are not shown. Genes are assigned to functional modules based on their primary annotated function; some genes (e.g., dnaK, which functions in both protein folding and stress tolerance) participate in multiple cellular processes and their module assignment reflects the predominant functional context discussed in this study.

To gain insight into the cellular responses associated with the enhanced oxidative stress tolerance observed in the RS16760-expressing strain, comparative RNA-seq analysis was performed between *E. coli* pET41a-RS16760 and the empty-vector control *E. coli* pET41a. Using the thresholds of |log_2_fold change| ≥ 1 and adjusted *p*-value ≤ 0.05, a total of 137 differentially expressed genes (DEGs) were identified, including 79 up-regulated and 58 down-regulated genes in *E. coli* pET41a-RS16760 relative to the control (Figure 5A). The two groups differed markedly in effect-size distribution: up-regulated genes exhibited predominantly moderate effect sizes (median log_2_FC = 1.53; 68.4% within 1 ≤ |log_2_FC | < 2), consistent with calibrated activation of the SoxRS regulon, whereas down-regulated genes showed substantially larger and more heterogeneous effect sizes (median log_2_FC = −2.12; 22.4% exceeding | log_2_FC | ≥ 4), dominated by near-complete repression of the tryptophan biosynthesis operon (*trp*A-E, log_2_FC = −7.9 to −12.3). The complete effect-size distribution and the 15 DEGs with | log_2_FC | ≥ 4 are detailed in Supplementary Figure S2 and Table S3, respectively.

Among the up-regulated DEGs, multiple genes associated with the SoxRS oxidative stress response and the heat-shock proteostasis network were prominently induced (Figure 5A,B). The oxidative stress module included *sox*S, *sod*A, *ahp*C, *grx*A, *zwf*, *fpr*, and *nfo*, encompassing a SoxRS-associated transcriptional regulator, superoxide dismutase, alkyl hydroperoxide reductase, glutaredoxin, and components of NADPH-regenerating and DNA repair systems. The heat-shock module included *ibp*A, *ibp*B, *dna*K, *dna*J, *htp*G, *hsl*U, and *hsl*V, consistent with a proteostatic response to heterologous overexpression burden. In parallel, *gln*A, encoding glutamine synthetase, was significantly up-regulated. Given the predicted L-Gln preference of the MWM45_RS16760 A domain, this transcriptional pattern is consistent with a substrate-supply feedback response, paralleling reports that nitrogen flux and glutamine availability are key constraints on indigoidine titres in heterologous hosts [10,42]. Direct confirmation of this interpretation would require measurement of intracellular glutamine pools, which is beyond the scope of the present study. These up-regulated patterns were further supported by GO enrichment analysis, which highlighted “response to heat”, “unfolded protein binding”, “HslUV protease complex”, “zinc ion binding”, and several redox-related molecular functions (Figure 5C).

Among the down-regulated DEGs, the tryptophan biosynthesis operon (*trp*A, *trp*B, *trp*CF, *trp*D, *trp*E) and the flagellar assembly genes (*flg*B, *flg*C, *flg*D, *flg*E) were concertedly repressed (Figure 5A,B). GO and KEGG enrichment analyses confirmed the over-representation of “tryptophan biosynthetic process”, “anthranilate synthase activity”, “bacterial-type flagellum-dependent motility”, “flagellar assembly”, and glyoxylate/glycolate-related metabolic terms (Figure 5D,E). Up-regulated DEGs did not yield statistically significant KEGG pathway enrichment under the applied criteria.

Collectively, these results reveal that heterologous expression of MWM45_RS16760 in *E. coli* is associated with coordinated transcriptional reprogramming, characterized by coordinated activation of oxidative-stress and proteostasis responses alongside concerted repression of tryptophan biosynthesis and flagellar assembly, suggesting that the enhanced oxidative stress tolerance of the RS16760-expressing strain is accompanied by both stress-responsive transcriptional remodeling and broader metabolic reallocation in the heterologous host.

## 4. Discussion

Water-soluble blue microbial pigments with documented antioxidant activity remain a comparatively under-represented class, and most mechanistic studies of pigment-mediated protection have focused on cell-free radical-scavenging chemistry rather than on host-level transcriptional responses [43,44]. The present work combined heavy-ion mutagenesis, comparative genomics, phylogenetic placement, heterologous reconstitution and comparative transcriptomics to identify MWM45_RS16760 as a candidate indigoidine-like NRPS in the glacier-derived strain *A. antioxidans* QL17. The data link blue-pigment yield to H_2_O_2_ tolerance across the wild-type strain and two pigmentation-altered derivatives and, after transfer to *E. coli*, show that the gene alone is sufficient to reconstruct a pigmented phenotype and is associated with enhanced oxidative-stress tolerance in the heterologous host. These observations are consistent with a dual-tier mode of protection that may combine direct extracellular radical scavenging with concerted transcriptional reprogramming of the host.

The canonical architecture of characterized indigoidine synthetases comprises a single A-Ox-PCP-TE module, in which the Ox domain installs the conjugated double bond that defines the chromophore. Pang et al. showed through in vitro reconstitution and site-directed mutagenesis that the Ox domain of BpsA uses Tyr665 as a general base to deprotonate the PCP-tethered L-glutaminyl intermediate, and proposed two parallel maturation routes for indigoidine biosynthesis [45]. Subsequent structural work on the bacillamide system has shown that an equivalent Tyr–FMN catalytic machinery can be supplied in trans by a discretely encoded oxidase, indicating that NRPS oxidation activity need not be embedded within the core module [46]. Against this backdrop, AlphaFold2 modeling and NRPSPredictor2 analysis of MWM45_RS16760 returned an apparent A-PCP-TE organization without a recognizable Ox domain. Two non-exclusive interpretations may be considered: oxidation activity could plausibly be supplied in trans by a host-encoded partner, paralleling the BmdB–BmdC arrangement in which a discretely encoded FMN-dependent oxidase acts co-synthetically with the NRPS [42], or the reported non-enzymatic interconversion of indigoidine and its hydrolysis products under aerobic conditions may contribute to chromophore maturation once the cyclic glutaminyl intermediate has been released by the TE domain [47]. Pang et al. (2020) demonstrated that the BpsA Ox domain uses Tyr665 as a catalytic base to deprotonate the PCP-tethered L-glutaminyl intermediate; whether analogous chemistry can be supplied by endogenous *E. coli* oxidoreductases remains to be determined [45]. The successful reconstitution of blue pigmentation in *E. coli* without co-expression of a dedicated oxidase (Figure 4A) suggests that endogenous oxidative capacity and/or spontaneous aerobic oxidation is sufficient for at least partial chromophore formation. Nevertheless, the absence of a canonical Ox domain in MWM45_RS16760 is noteworthy, as characterized indigoidine synthetases often contain an oxidation domain involved in chromophore formation. However, because no direct biochemical or oxidation-state analysis was performed in this study, the mechanistic implications of this domain architecture should be interpreted with caution. It may indicate an Ox-independent route or the involvement of other oxidative processes, but these possibilities remain speculative and require further experimental validation. Phylogenetically, the A domain of MWM45_RS16760 clustered with *Pseudarthrobacter*-derived sequences in a well-supported subclade (UFBoot = 100) separated from both the *Streptomyces*-associated BpsA branch and the *Dickeya*-associated IndC branch. Pigmented *Arthrobacter* and *Pseudarthrobacter* isolates are widespread in glaciers and alpine soils [48], but their NRPS-derived metabolic potential has been sparsely tested, in part because many actinobacterial BGCs remain cryptic under standard cultivation [49]. To our knowledge, no experimentally characterized indigoidine synthase has previously been reported from this lineage.

Interest in the redox chemistry of indigoidine dates back to the observation that the pigment is bleached upon hydroxyl-radical capture and improves H_2_O_2_ tolerance in indigoidine-overproducing strains [31]. The fully conjugated bipyridyl skeleton, intramolecular hydrogen bonding and narrow band gap that underlie this reactivity [8] are compatible with the dual SET/HAT operating modes invoked for the DPPH and ABTS assays [9]. Quantitatively, microbial pigments span a broad activity range: prodigiosin from cold-adapted *Janthinobacterium* ERMR3:09 [50] and from *S. marcescens* VITSD2 [51] shows dose-dependent DPPH scavenging at the milligram per milliliter scale, whereas arthroxanthin from the same *A. antioxidans* QL17 parental strain has been characterized with DPPH and ABTS IC_50_ values of 69.8 and 21.5 µg/mL, respectively [19]. In the present study, the culture supernatant of *E. coli* pET41a-RS16760 scavenged approximately 55% of DPPH and 42% of ABTS radicals, both values significantly above the empty-vector control. These figures describe the bulk activity of unfractionated supernatant rather than the intrinsic specific activity of a purified pigment, and the chemical identity of the responsible chromophore has not been directly verified here; they should therefore be interpreted accordingly. We cannot exclude the possibility that other metabolites co-secreted by the RS16760-expressing strain, including potential indigoidine hydrolysis products or unrelated *E. coli* metabolites induced by heterologous expression, contribute to the observed scavenging activity. Attribution of the antioxidant activity to the blue chromophore specifically awaits fractionation and activity-guided purification. The biological relevance of an extracellular scavenging capacity nevertheless aligns with the principle that interception of oxidants before they reach intracellular Fenton-active iron pools is among the more efficient protective strategies available to aerobic cells [52].

Beyond direct scavenging, the transcriptional profile points to a coordinated host-side response. The SoxRS-associated oxidative-stress response is triggered by superoxide and nitric oxide through oxidation of the SoxR [2Fe–2S] cluster, with SoxS activating downstream effectors such as *sod*A, *zwf*, *fpr* and *nfo* [53]; NADPH depletion alone can elicit the response in the absence of measurable ROS accumulation [54], and rapid induction of *sod*A and *zwf* occurs within minutes of an oxygen up-shift [55]. The RNA-seq profile reported here recapitulates this core set, with *sox*S, *sod*A, *ahp*C, *grx*A, *zwf*, *fpr* and *nfo* all significantly up-regulated in the RS16760-expressing strain. The concurrent induction of *ahp*C, which is primarily under OxyR control, suggests that SoxRS- and OxyR-associated responses may be jointly elevated, in line with the integrated view of peroxide and superoxide stress [44]. This integrated view resonates with the broader ROS/RNS signaling framework increasingly recognized across aerobic organisms [56]. An intriguing mechanistic possibility is that the pigment itself, or a biosynthetic intermediate, may serve as the proximate SoxR oxidant. Gu and Imlay [57] demonstrated that the SoxR [2Fe–2S] cluster is directly oxidized by redox-cycling compounds rather than by superoxide per se, with the solvent-exposed [2Fe–2S] cluster enabling rapid electron transfer with diverse redox-active agents [58]. The fully conjugated bipyridyl chromophore of indigoidine possesses a narrow HOMO–LUMO gap and documented electron-transfer capacity [8], properties that are, in principle, compatible with one-electron oxidation of the SoxR [2Fe–2S]^1+^ cluster. Alternatively, PCP-tethered glutaminyl intermediates generated during NRPS catalysis may act as transient intracellular redox-active species capable of perturbing the SoxR redox poise, analogous to the redox-cycling drug paradigm [59]. Either scenario would establish a direct mechanistic link between pigment biosynthesis and the transcriptional arm of oxidative defense, unifying the two protective tiers—extracellular radical scavenging and intracellular transcriptional reprogramming—proposed in this study. Distinguishing between these possibilities will require in vitro reconstitution of the SoxR–pigment interaction, an important objective for future work.

The proteostasis module showed coordinated up-regulation of *ibp*A, *ibp*B, *dna*K, *dna*J, *htp*G, *hsl*U and *hsl*V. Earlier work reported that IbpA/B overexpression confers resistance to both heat and superoxide stress [60], and IbpA/B are now understood to act as ATP-independent holdases that sequester partially denatured substrates prior to transfer to the DnaK/ClpB chaperone system [61]. Two non-exclusive interpretations are compatible with the data: a generic σ32-mediated burden imposed by heterologous NRPS overexpression, and a more specific recruitment of chaperone capacity in support of oxidative defense. The two mechanisms may jointly contribute to the right-shifted H_2_O_2_ survival curve observed in *E. coli* pET41a-RS16760. Of note, *gln*A encoding glutamine synthetase was also significantly up-regulated; given the predicted L-Gln preference of the MWM45_RS16760 A domain, this pattern is consistent with a substrate-supply feedback, paralleling reports that nitrogen flux is a key constraint on indigoidine titres in heterologous hosts [10,42].

The transcriptional response also included coordinated repression of two functionally coherent modules. The tryptophan biosynthesis operon (*trp*A, *trp*B, *trp*CF, *trp*D, *trp*E) was repressed as a group. Two non-exclusive explanations may account for this pattern. First, the Trp indole side chain is readily oxidized to N-formylkynurenine, kynurenine, and tryptophan hydroperoxide intermediates with measurable consequences for protein function [62,63], so coordinated down-regulation of de novo Trp synthesis is consistent with a metabolic adjustment that may limit the supply of an oxidation-prone amino acid under sustained oxidative stress. Second, the strong up-regulation of *zwf*, encoding glucose-6-phosphate dehydrogenase in the oxidative branch of the pentose phosphate pathway (PPP), indicates an increased cellular demand for NADPH to support the glutathione- and thioredoxin-based antioxidant systems. In *E. coli*, the shikimate pathway-from which Trp biosynthesis branches at the chorismate node-draws erythrose 4-phosphate from the non-oxidative branch of the PPP [64,65]. Thus, the concurrent suppression of Trp biosynthesis may also reflect metabolic re-routing to prioritize NADPH regeneration via the oxidative PPP at the expense of the chorismate-dependent aromatic amino acid pathway. These two mechanisms—reducing oxidation-sensitive substrates and re-allocating carbon toward NADPH production—are not mutually exclusive and may jointly contribute to the observed transcriptional pattern. Similarly, the flagellar assembly genes *flg*B, *flg*C, *flg*D and *flg*E were repressed. Flagellar biosynthesis and rotation are among the more resource-intensive processes in *E. coli*, consuming a substantial share of the protein and energy budgets and competing for proton-motive force [66]; at the single-cell level, *fli*C-OFF Salmonella tolerate antibiotics better than *fli*C-ON counterparts, a trade-off attributed to PMF competition with TolC-dependent efflux [67]. The coordinated flagellar repression observed here is therefore consistent with reallocation of cellular resources from motility towards stress responses.

Several limitations should be acknowledged. Heterologous BpsA and IndC systems typically require co-expression of a B. subtilis Sfp-type PPTase to convert apo-PCP to holo-PCP [42]; the present construct relied on endogenous *E. coli* EntD, and although the resulting pigmentation indicates at least partial compatibility, whether Sfp co-expression would raise titres remains to be tested. In addition, heterologous expression of MWM45_RS16760 and pigment biosynthesis may impose additional metabolic and proteostasis burdens on *E. coli*. Therefore, the observed transcriptomic changes should be interpreted as host responses associated with RS16760 expression and pigment production, rather than as direct evidence of pigment-specific regulatory effects. The pigment was characterized only by UV-Vis absorption in this study. A parallel study (manuscript under review) has elucidated the structure of the native blue pigment from *A. antioxidans* QL17 by NMR and HRMS, and has revealed that its complete biosynthesis involves seven genes within the Cluster 7 BGC, of which MWM45_RS16760 is the core biosynthetic gene. Consequently, heterologous expression of RS16760 alone in *E. coli* is expected to yield a core biosynthetic product that may differ structurally from the fully mature native pigment, which likely requires additional tailoring, transport, or regulatory functions encoded by the remaining cluster genes. The present study was designed to establish the functional role of RS16760 in blue pigment production and oxidative stress resistance rather than to characterize the chemical identity of its heterologous product. Future work will include isolation and structural identification of the RS16760-derived blue pigment by NMR and HRMS, and direct comparison with the native QL17 pigment to determine the extent of structural equivalence and to establish structure–activity relationships. In vitro enzymatic reconstitution and clean knockout-complementation in *A. antioxidans* QL17 will likewise be needed to consolidate causal attribution. Although off-target mutations introduced by heavy-ion irradiation cannot be excluded without whole-genome re-sequencing of M157 and M186, the concordance between the three-strain pigment-tolerance correlation, the matching RT-qPCR expression pattern, and the independent reconstitution of both phenotypes in *E. coli* by MWM45_RS16760 alone collectively minimize the likelihood that off-target effects account for the observed association. Comparison with high-titre *Streptomyces* [68] and *P. putida* [62] platforms also indicates considerable headroom for process engineering, and supports further evaluation of MWM45_RS16760 as a candidate genetic target for engineering antioxidant-enhanced microorganisms with potential food, cosmetic and pharmaceutical applications.

## 5. Conclusions

In this study, we identified and characterized MWM45_RS16760, a novel indigoidine-like nonribosomal peptide synthetase (NRPS) gene from the glacier-derived strain *A. antioxidans* QL17, and assessed its role in oxidative stress resistance through an integrated framework combining heavy-ion mutagenesis, genome mining, phylogenetic reconstruction, heterologous expression, and comparative transcriptomics. Pigmentation-altered mutants demonstrated that blue pigment yield was positively associated with hydrogen peroxide (H_2_O_2_) tolerance in *A. antioxidans* QL17, and transcript-level analysis across the wild-type and mutant strains supported MWM45_RS16760 as the candidate core biosynthetic gene. The encoded enzyme adopts an adenylation-peptidyl carrier protein-thioesterase (A-PCP-TE) architecture with a predicted L-glutamine-preferring A domain and is phylogenetically placed within a distinct actinomycete-associated indigoidine-like lineage separated from the characterized BpsA and IndC branches, expanding the known taxonomic and structural diversity of indigoidine-like biosynthetic enzymes. Heterologous expression in *E. coli* reconstructed a blue-pigment-producing phenotype and enhanced H_2_O_2_ tolerance together with elevated extracellular DPPH and ABTS radical-scavenging activities, while comparative transcriptomics revealed that this protective phenotype was accompanied by coordinated induction of SoxRS-associated oxidative-stress responses and the heat-shock proteostasis network alongside concerted repression of tryptophan biosynthesis and flagellar assembly. Collectively, these findings support MWM45_RS16760 as a candidate indigoidine-like NRPS associated with both blue pigment biosynthesis and oxidative-stress resistance, pending biochemical validation of the pigment identity, in vitro enzymatic reconstitution, and yield optimization. MWM45_RS16760 may warrant further investigation as a genetic target for engineering antioxidant-enhanced microorganisms for potential food, biomedical, and cosmetic applications.

## Data Availability

All data supporting the findings of this study are included in this published article and its Supplementary Materials. The raw sequencing data presented in this study are openly available in SRA at accession number PRJNA1468948. The original data presented in the study are included in the article/Supplementary Materials. Further inquiries can be directed to the corresponding author.

## Supplementary Materials

The following supporting information can be downloaded at: https://www.mdpi.com/article/10.3390/antiox15070846/s1, Figure S1: Complete maximum-likelihood phylogenetic tree of the adenylation (A) domain of MWM45_RS16760 and bacterial homologs; Figure S2: Distribution of effect sizes (log_2_FC) among the 137 DEGs identified between *E. coli* pET41a-RS16760 and *E. coli* pET41a; Table S1: Primers used for RT-qPCR analysis; Table S2: Binding affinities from triplicate molecular docking of L-glutamine to the adenylation domain of MWM45_RS16760; Table S3: Differentially expressed genes with |log*2*FC| ≥ 4 between *E. coli* pET41a-RS16760 and *E. coli* pET41a.
