# Supplementary material for: A Novel Indigoidine-like NRPS Gene from Arthrobacter antioxidans QL17 Enhances Oxidative Stress Resistance Through Radical Scavenging and Transcriptional Reprogramming
Source: Antioxidants (Basel). 2026 Jul 4;15(7):846. doi: 10.3390/antiox15070846 (PMC13405713; doi:10.3390/antiox15070846)
Supplement: Supplementary file 1 [file antioxidants-15-00846-s001.zip › antioxidants-4362134-supplementary.pdf]

Table S1. Primers used for RT-qPCR analysis.

Primer Name	Sequence (5'→3')	Purpose
RS16760-F	GCTGATGTGTTGCGATGGAT	Target gene (RS16760)
RS16760-R	ATGAAGGCGAAGGCTTGGA	Target gene (RS16760)
16S rRNA-F	GCTGTAACTGACGCTGAGGA	Reference gene
16S rRNA-R	CACAAGTGGCTGACACATCTC	Reference gene

Table S2. Comparative molecular docking of L-glutamine and five control amino acids to the adenylation domain of MWM45_RS16760.

Amino acid	ΔG mean ± SD (n=3, kcal/mol)	Pocket contacts (≤ 3.5 Å)	Contacting residues	p vs L-Gln
L-Gln	−5.858 ± 0.014	4	Thr198, Gln245, Gly272(×2)	—
L-Glu	−5.720 ± 0.020	4	Thr198, Gln245, Gly272	0.00114
L-Asn	−5.418 ± 0.004	3	Gln245, Gly272(×2)	0.00012
L-Lys	−5.669 ± 0.111	1	Gly272	0.09534
L-Ala	−4.230 ± 0.004	0	—	<0.00001
L-Phe	−6.648 ± 0.012	0	—	<0.00001

Table note: P-values from Welch's two-sample t-test (unequal variance), comparing each ligand's triplicate binding affinities to L-Gln (n=3 per group). Polar contacts defined as N/O···N/O distances ≤3.5 Å with the four PARAS-identified substrate-recognition residues (Thr198, Ala204, Gln245, Gly272).

Table S3. Differentially expressed genes with $|\log_2FC| \geqslant 4$ between *E. coli* pET41a-RS16760 and *E. coli* pET41a. A total of 15 genes (2 up-regulated, 13 down-regulated) exceeded the very-large effect-size threshold. padj, Benjamini–Hochberg FDR-adjusted P-value.

Gene	Log_2FC	Adjusted P-value	Direction
trpA	-12.32	8.89×10^{-22}	down
trpB	-12.26	1.30×10^{-21}	down
HO396_06465	-11.75	4.18×10^{-20}	down
trpCF	-10.60	4.83×10^{-16}	down
HO396_06380	-10.54	1.25×10^{-15}	down
yciV	-10.10	2.58×10^{-14}	down
HO396_06400	-9.89	5.70×10^{-14}	down
yciA	-9.58	6.57×10^{-13}	down
trpD	-9.08	1.16×10^{-11}	down
HO396_06395	-8.93	3.97×10^{-15}	down
trpE	-7.88	1.88×10^{-08}	down
tonB	-7.78	2.23×10^{-08}	down
HO396_06415	-6.89	8.78×10^{-06}	down
soxS	4.95	4.59×10^{-54}	up
nifJ	4.06	3.15×10^{-23}	up

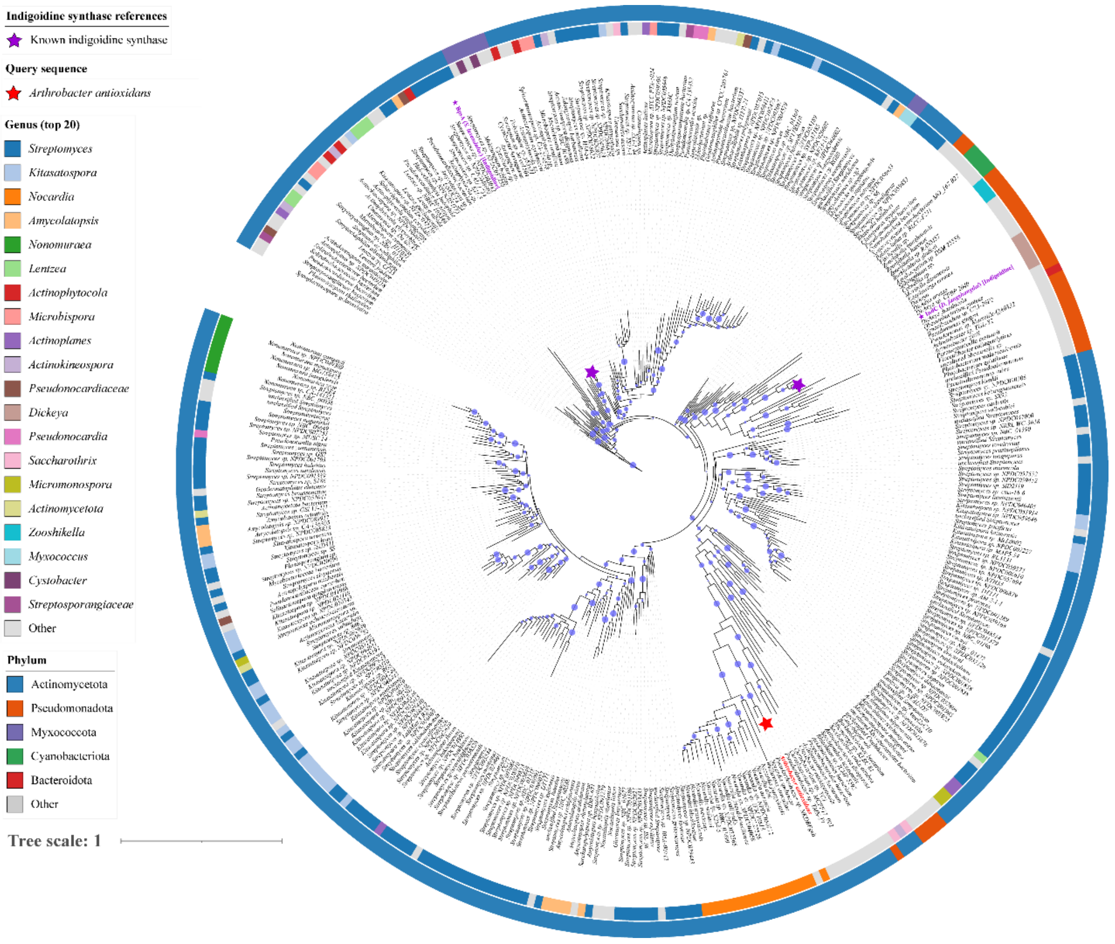

Figure S1. Complete maximum-likelihood phylogenetic tree of the adenylation (A) domain of MWM45_RS16760 and bacterial homologs.

A maximum-likelihood phylogenetic tree was reconstructed from the A domain sequences of MWM45_RS16760 (Arthrobacter antioxidans QL17) and 370 bacterial homologs retrieved from the NCBI non-redundant protein database, together with two reference indigoidine synthases: BpsA from Streptomyces lavendulae and IndC from Dickeya fangzhongdai. The query sequence is marked with a red star , and the two reference indigoidine synthases are marked with purple stars. Ultrafast bootstrap (UFBoot) support values (⩾ 80%) are indicated by blue circles at internal nodes. The inner and outer colored strips annotate taxonomic affiliation at the genus and phylum levels, respectively.

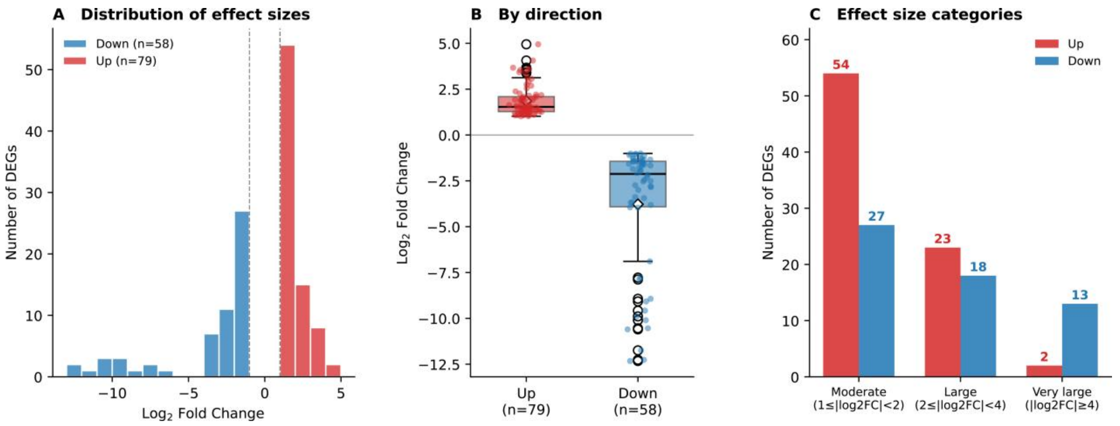

Figure S2. Distribution of effect sizes ($\log_2$FC) among the 137 DEGs identified between *E. coli* pET41a-RS16760 and *E. coli* pET41a. (A) Histogram of $\log_2$FC values stratified by direction of regulation (up, red; down, blue). Dashed lines indicate the $|\log_2 FC| \geqslant 1$ threshold. (B) Boxplot comparing $\log_2$FC values between up-regulated (n = 79) and down-regulated (n = 58) DEGs. Horizontal lines indicate medians; diamonds indicate means; circles indicate outliers. (C) Number of DEGs in three effect-size categories: moderate ($1 \leqslant |\log_2 FC| < 2$), large ($2 \leqslant |\log_2 FC| < 4$), and very large ($|\log_2 FC| \geqslant 4$), stratified by direction. Numbers above bars indicate gene counts.
